# Supplementary material for: Archaeological and molecular evidence for ancient chickens in Central Asia
Source: Nat Commun. 2024 Apr 2;15:2697. doi: 10.1038/s41467-024-46093-2 (PMC10987595; doi:10.1038/s41467-024-46093-2)
Supplement: Supplementary file 1 — Supplementary Information [file 41467_2024_46093_MOESM1_ESM.pdf]

Archaeological and molecular evidence for ancient chickens in Central Asia

Carli Peters; Kristine K. Richter; Shevan Wilkin; Sören Stark; Basira Mir-Makhamad; Ricardo Fernandes; Farhad Maksudov; Mirzaakhmedov Sirojidin; Husniddin Rahmonov; Stefanie Schirmer; Kseniia Ashastina; Alisher Begmatov; Michael Frachetti; Sharof Kurbanov; Michael Shenkar; Taylor Hermes; Fiona Kidd; Andrey Omelchenko; Barbara Huber; Nicole Boivin; Shujing Wang; Pavel Lurje; Madelynn von Baeyer; Rita Dal Martello; and Robert N. Spengler III

This PDF file includes:

Supplementary Discussion: The Chicken Domestication Debate
Supplementary Table 1: Sample numbers, site and context information, and final identification of the eggshell fragments included in this study.
Supplementary Table 2: Taxonomy of Phasianidae in Central Asian and neighboring regions
Supplementary References

Supplementary Discussion: Sites Containing Eggshell Discussed in this Manuscript

Bash Tepa
Bash Tepa is a Hellenistic fortified site, possibly a citadel that was once surrounded by agricultural land and likely small residential farmsteads that has now been deflated by wind. Presumably, occupation within the fortifications would have been restricted to more elite members of the population, but the excavators suggest that the social function of the fortifications may have changed over time[1]. Later occupations consisted of pit-house structures built into the top of the mound site, and Stark et al.[2] noted that the dates primarily fall, with a probability of 95% (2σ), between 350 and 50 BC. Ceramics and other material culture attest to local-scale exchange and interaction, notably within Sogdiana, but also with Hellenistic Bactria and Khoresam[1,2]. Eggshell fragments were found across all tested contexts at the site, including pithouses dug into the main site structure. The fill in these pithouses likely originated away from the site center, but consist of cultural sediments.

Paykend
Paykend was a major urban hub on the ancient trade routes and likely served as a center of commerce on the edge of the Bukhara Oasis. The site complex went through numerous social and functional changes over time, and we, therefore, have divided the complex into three functionally and temporally distinct components. Sediment samples were collected from many cultural layers at Paykend. The earliest clear occupation levels at the site (Paykend 1) consist of a fortified citadel similar to and roughly contemporaneous with that of Bash Tepa, but larger in area. Two samples were collected from the earliest cultural layers, dating to the 3rd-2nd centuries BC. Eleven eggshells were recovered in one of two samples coming from the area near the citadel with a total density of 0.23 eggshell fragments per liter of sediment. In addition to samples from the Hellenistic period, five samples were taken from the cultural layers dating to the 3rd-4th centuries AD (also Paykend 1). The Paykend 2 samples have been dated to the Qarakhanid period coming from the Shakhristan II, roughly contemporaneous with the one sampled at Afrasiab. Eggshell were present in only two of these samples. We also collected 20 sediment samples from Rabat-4 at Paykend 3 dated to the 10th-12th centuries AD (the Qarakahanid period). The use of this rabat or caravansary appears to continue after

the abandonment of the neighboring urban center, and likely continued to serve as a rest stop for merchant caravans. The ubiquity of eggshells in these was 45 percent (9 out 20 samples in Paykend 3). Eggshells in Paykend 3 (Rabat) were recovered only in the samples taking from cooking/tandyr ovens.

Burial LVD-HA-K7

Burial mound LVD-HA-K7 was recently excavated in the Kyzyl Kum Desert, on the edge of the Bukhara Oasis, and eggs appear to have been part of the mortuary offerings. A small pile of eggshells was recovered from inside the burial chamber, in the southeastern corner, among ceramics (bowls, goblets, etc.) and other evidence for food offerings. Interestingly, a few decades earlier, a pile of eggshells was recovered from another burial in the same are, burial HZR-K7, and the eggshells were referred to as chicken and found in association with shellfish. Archaeological eggshell finds from Dabusia (located halfway between Bukhara and Samarkand) and Kurgans in Fergana have also been reported[3]. The offerings were recovered from close to the elbow of a human skeleton[4]. Both contexts have been identified as nomadic kurgans or burial mounds and thought to belong to a different people from those who occupied the oasis cities of Bukhara, such as Paykend and Bukhara proper. Interestingly, the eggshell fragment from LVD-HA-K7 turned out to be one of the two tested fragments that were not from chicken and instead appear to be from a waterfowl. We assume that they represent an example of people collecting wild migratory bird eggs as the birds passed through the marshes of the Bukhara Oasis.

Panjakent

Ancient Panjakent is a well-known Sogdian city located in western Tajikistan on the southern bank of the Zarafshan River. The Sogdian city emerged in the 5th century AD and remained a prosperous urban center until the 8th century AD and was abandoned after the Arabic Expansions. The Panjakent site consists of two distinct parts: the main city Panjakent and citadel on a separate hill with the spring of Kainar underneath. It probably emerged several centuries earlier. In total, 81 eggshell fragments were recovered during the flotation of samples from the citadel (n=25), and in the main city (n=56) the shells predominantly came from Objects VIC and XII-B. Density of eggshell fragments per liter of sediment was 0.2 in both the citadel and the main city.

Kafir Kala

The large fortified tell site of Kafir Kala is located 12 kilometers south of the modern city of Samarkand. The fortifications have been used since at least the 4th century AD and continued to be occupied through the Qarakhanid period. Eggshells were handpicked from the site during the 2020 excavations and date to the Samanid period, likely in the eight century AD.

Ming Tepa

Ming Tepa is located about 20 km northeast of Afrasiab. This site is thought to be identified with Kabudhan, a late antique and early medieval Sogdian city, attested in Chinese and Arabic sources. The eggshells from the site were handpicked and not systematically collected from wet screening. The shells are thought to belong to the layers of 7th - 8th centuries AD[5]. Other handpicked eggshells from archaeological sites in Central Asia have been reported, such as from an early medieval site in Ferghana[3].

Sanjar-Shah

Sanjar-Shah is located 12 km to the east of Panjakent in western Tajikistan. Sanjar-Shah is the largest settlement in the neighborhood of Panjakent; moreover, the city was likely part of the city-state of Panjakent[6]. The occupation layers dated preliminarily to the 5 – 9th centuries AD in Sanjar-Shah. In 2021, five sediment samples were taken and floated. A total of 65.5 L of floated sediment yielded 26 eggshell fragments with a density of 0.39 eggshell fragments per liter of sediment. Eggshell fragments were present in four out of five samples. The majority of eggshells (n=19) came from the sample that represents the tandyr filling.

Kuk-Tosh

Kuk-Tosh, also known as pre-Mongol Panjakent, is located in the eastern portion of the modern city of Panjakent and was occupied from the Samanid to the Qarakhanid periods (roughly 9 – 12th centuries AD). Based on cultural artifacts, notably glass[7] and ceramics[8], Kuk-Tosh inhabitants took an active role in expanding trade and economic ties in Panjakent. During the pilot archaeobotanical study, 2,847 eggshell fragments were recovered, almost 99.6% of those eggshells came from the cesspit deposit.

Bukhara

Rescue excavations have been carried out just to the east of the citadel (the Arc) of medieval Bukhara, one of the two capital cities of the Qarakhanid Empire (along with Afrasiab – also in this study). The excavated area may either have represented an elite residential neighborhood or a market bazaar. Radiocarbon dates, and material culture remains both securely place occupation of the area around the end of the Samanid and through the Qarakhanid phases, from the ninth through the eleventh centuries AD. Within which, 871 eggshell fragments were recovered. These shell fragments have been recovered both from cesspits and sediment layers, suggesting that eggs were prominent in the diet.

Tashbulak

Tashbulak is a village or urban site located at high elevation (2200masl) in the Pamir Mountains of Uzbekistan, and was excavated over several years from 2016 through 2018[9]. An extensive radiocarbon dating campaign, confirmed by coins found at the site, places occupation around the late tenth century. Eggshell fragments were recovered from 5 of 22 heavy fraction samples from the Tashbulak site, totaling 95 fragments; although, 86 of those fragments came from one sample (FS26). Two excavation units were sampled, one in the citadel at the site, which contained a single shell fragment and the other from a series of contemporaneous strata in an adjacent midden, where the remaining fragments were recovered. It is worth noting that these fragments come from multiple flotation samples collected from different parts of the site, including multiple depositional layers in the central midden.

Afrasiab

Afrasiab was permanently inhabited from pre-Achaemenid periods until 1221; it was a capital of Sogdiana and a central hub on the ancient Silk Road trade routes. In the last centuries of its existence it was one of two capitals (along with Bukhara) of the Samanid and Qarakhanid states. One sediment sample with a total volume of 255 L has been collected from a midden within a domestic context, approximately 500 m from the Afrasiab citadel in 2019. Three ancient grains were selected from the midden for radiocarbon dating and they range from 800 to 1100, confirming the assumptions of the excavators based on material culture. The occupation area where the sample was collected would have represented economically prosperous members of the empire living in the core of its capital, but not elite enough to

reside in the palace residence itself. Eggshells were abundant in this assemblage; in total 855 eggshell fragments have been recovered from the heavy fraction with a density of 3.35 eggshell fragments per liter. Seven eggshells out of 855 showed signs of carbonization.

Supplementary Table 1: Sample numbers, site and context information, and final identification of the eggshell fragments included in this study.

**Sample number**	**Site**	**Context**	**Identification**
KKX1000	Burial Mound LVD-HA-K7	Burial chamber	cf. Anseriformes
KKX1001	Bash Tepa	FS10	*Gallus gallus*
KKX1002	Bash Tepa	FS13	*Gallus gallus*
KKX1003	Tashbulak		*Gallus gallus*
CP564	Afrasiab	FSA1 "F"	*Gallus gallus*
CP565	Afrasiab	FSA1 "D"	*Gallus gallus*
CP566	Afrasiab	FSA1 "A"	*Gallus gallus*
CP567	Afrasiab	FSA1 "C"	*Gallus gallus*
CP568	Afrasiab	FSA1 "E"	*Gallus gallus*
CP569	Paykend	FS8A	*Gallus gallus*
CP570	Paykend	FS8B	*Gallus gallus*
CP571	Paykend	FS21	*Gallus gallus*
CP572	Paykend	FS15	*Gallus gallus*
CP573	Paykend	FS29	*Gallus gallus*
CP574	Bash Tepa	18FS3HF	*Gallus gallus*
CP575	Bash Tepa	18FS1HF	*Gallus gallus*
CP576	Bash Tepa	17FS6HF	*Gallus gallus*
CP577	Burial Mound LVD-HA-K7	FS1A	Anseriformes

Supplementary Table 2: Taxonomy of Phasianidae in Central Asian and neighboring regions.

**Phasianidae (Central Asian and Peripheral Species)**					
**Rollulinae**	**Phasianinae**				
None Present in Central Asia	Galini	Phasianini	Tetraonini	Coturnicini	Lophophorini
	None Present in Central Asia other than the Domesticated Chicken	*Phasianus colchicus*	*Tetrastes bonaisia*	*Coturnix coturnix*	*Tetraophasis spp.*
		Francolinus francolinus	*Tetrao urogalloides*	*Alectoris chukar*	*Lophopharus spp.*
		Crateus wallichii	*Lyrurus tetrix*	*Pterocles gutturalis*	
		Crossoptilon harmani	*Perdix dauurica*		
			Perdix hodgsoniae		

Note that species present in Central Asia (loosely defined here to include all arid and mountainous regions) are highlighted in green; species present in the peripheral mountainous areas of western China are left un-highlighted. While species that are peripheral to Central Asia are highly unlikely to have been targeted for their eggs by ancient peoples, we mark them here in order to remain conservative in all our discussions.

Supplementary References

1 Stark, S. *et al.* The Uzbek-American Expedition in Bukhara. Preliminary Report on the Third Season (2017). *Iran*, 1-51, doi:10.1080/05786967.2020.1769495 (2020).

2 Stark, S. *et al.* Bashtepa 2016: Preliminary Report of the First Season of Excavations. *Archäologische Mitteilungen aus Iran und Turan* **48**, 219-264 (2016).

3 Gorbunova, N.H. The culture of ancient Ferghana: VI century B.C. – VI century A.D. (BAR, Oxford, 1986).

4 Obel'chenko, O. V. *Kul'tura Antichnogo Sogda: Po Arkheologicheskim Dannym VII v. do n.e.- VII v. n.e.* (Main Editorial Office of Eastern Literature, Moscow, 1992).

5 Begmatov, A. *et al.* The formation and development of cities in the oases of Central Asia: excavations at Ming Tepa in Uzbekistan. *Proc. 29th Annu. Meeting Excavations West Asia. Jpn. Soc. West Asian Archaeol.*, 18-22 (2021).

6 Kurbanov, S. in *Urban Cultures of Central Asia from the Bronze Age to the Karakhanids* Vol. 12 (eds C. Baumer & M. Novák) (Harrassowitz Verlag, Wiesbaden, 2019).

7 Aminov, F. Стеклянные изделия домонгольского Пенджикента и его округи (Pre-Mongolian Glass from Panjakent and its suburbs). *Transactions of the Institute of History of Material Culture of the Russian Academy of Sciences* **23**, 103-118, doi:10.31600/2310-6557-2020-23-103-118 (2020).

8 Aminov, F. in *Традиция государства Саманидов и ее воздействие на культуры и цивилизации народов Центральной Азии и Ближнего Востока* 68-72 (TNU, Dushanbe, 2020).

9 Maksudov, F. *et al.* in *Urban Cultures of Central Asia from the Bronze Age to the Karakhanids: Learnings and conclusions from new archaeological investigations and discoveries. Proceedings of the First International Congress on Central Asian Archaeology held at the University of Bern, 4–6 February 2016* 283-306 (Harrassowitz Verlag, Bern, Germany, 2019).
