# Supplementary material for: Archaeological and molecular evidence for ancient chickens in Central Asia
Source: Nat Commun. 2024 Apr 2;15:2697. doi: 10.1038/s41467-024-46093-2 (PMC10987595; doi:10.1038/s41467-024-46093-2)
Supplement: Supplementary file 3 — Description of Additional Supplementary Files [file 41467_2024_46093_MOESM3_ESM.pdf]

Supplementary Data 1: Key Archaeological Sites Supporting the Westward Spread, Linked to Figure 1

Supplementary Data 2: Overview of all sediment samples analysed and number of eggshells recovered from these samples.

Supplementary Data 3: Overview of all *m/z* peaks found in eggshell samples identified as chicken with peptide mass fingerprinting.

Supplementary Data 4: Proteins identified in sample CP565 (Afrasiab) using Byonic with a LogProb ≥ 5, and at least 2 PSMs having PEP2D scores <0.001.

Supplementary Data 5: Proteins identified in sample CP565 (Afrasiab) using Mascot.
