# Supplementary material for: Archaeological and molecular evidence for ancient chickens in Central Asia
Source: Nat Commun. 2024 Apr 2;15:2697. doi: 10.1038/s41467-024-46093-2 (PMC10987595; doi:10.1038/s41467-024-46093-2)
Supplement: Supplementary file 9 — Reporting Summary [file 41467_2024_46093_MOESM9_ESM.pdf]

nature portfolio

Corresponding author(s): Robert Spengler
Last updated by author(s): Nov 22, 2023

Reporting Summary

Nature Portfolio wishes to improve the reproducibility of the work that we publish. This form provides structure for consistency and transparency in reporting. For further information on Nature Portfolio policies, see our Editorial Policies and the Editorial Policy Checklist.

Statistics

For all statistical analyses, confirm that the following items are present in the figure legend, table legend, main text, or Methods section.

n/a	Confirmed	
☒	☐	The exact sample size (*n*) for each experimental group/condition, given as a discrete number and unit of measurement
☒	☐	A statement on whether measurements were taken from distinct samples or whether the same sample was measured repeatedly
☒	☐	The statistical test(s) used AND whether they are one- or two-sided<br>*Only common tests should be described solely by name; describe more complex techniques in the Methods section.*
☒	☐	A description of all covariates tested
☒	☐	A description of any assumptions or corrections, such as tests of normality and adjustment for multiple comparisons
☒	☐	A full description of the statistical parameters including central tendency (e.g. means) or other basic estimates (e.g. regression coefficient) AND variation (e.g. standard deviation) or associated estimates of uncertainty (e.g. confidence intervals)
☒	☐	For null hypothesis testing, the test statistic (e.g. *F*, *t*, *r*) with confidence intervals, effect sizes, degrees of freedom and *P* value noted<br>*Give P values as exact values whenever suitable.*
☒	☐	For Bayesian analysis, information on the choice of priors and Markov chain Monte Carlo settings
☒	☐	For hierarchical and complex designs, identification of the appropriate level for tests and full reporting of outcomes
☒	☐	Estimates of effect sizes (e.g. Cohen's *d*, Pearson's *r*), indicating how they were calculated

Our web collection on statistics for biologists contains articles on many of the points above.

Software and code

Policy information about availability of computer code

Data collection	No custom software or code was used in the data collection for this project.
Data analysis	No custom software of code was used in the data analysis for this project.

For manuscripts utilizing custom algorithms or software that are central to the research but not yet described in published literature, software must be made available to editors and reviewers. We strongly encourage code deposition in a community repository (e.g. GitHub). See the Nature Portfolio guidelines for submitting code & software for further information.

Data

Policy information about availability of data

All manuscripts must include a data availability statement. This statement should provide the following information, where applicable:

- Accession codes, unique identifiers, or web links for publicly available datasets
- A description of any restrictions on data availability
- For clinical datasets or third party data, please ensure that the statement adheres to our policy

The MALDI-ToF-MS spectra thatgenerated in support the findings of this study have been deposited onare available in Zenodo (doi: 10.5281/zenodo.4084517). The MS/MS data files are available on ProteomExchange under accession codeat PXD031493 and were uploaded through MassIVE (MSV000088794, doi: 10.25345/C5HK35).

Human research participants

Policy information about studies involving human research participants and Sex and Gender in Research.

Reporting on sex and gender	N/A
Population characteristics	N/A
Recruitment	N/A
Ethics oversight	N/A

Note that full information on the approval of the study protocol must also be provided in the manuscript.

Field-specific reporting

Please select the one below that is the best fit for your research. If you are not sure, read the appropriate sections before making your selection.

☐ Life sciences ☐ Behavioural & social sciences ☒ Ecological, evolutionary & environmental sciences

For a reference copy of the document with all sections, see nature.com/documents/nr-reporting-summary-flat.pdf

Ecological, evolutionary & environmental sciences study design

All studies must disclose on these points even when the disclosure is negative.

Study description	Palaeoproteomic analysis (using both peptide mass fingerprinting and shotgun proteomics) of archaeological eggshell fragments to identify which birds these eggshell fragments belong to. A total of 20 specimens were selected out of roughly 50,000 specimens to test for taxonomic identifications.
Research sample	All samples included in the study are archaeological eggshell fragments - the goal of the study was to illustrate that a prominent portion of the overall archaeological assemblage was chickens; although, we cannot exclude the possibility of other random species being present at lower prominences.
Sampling strategy	No statistical method was used to predetermine sample size. Sampling was done randomly among the large collection of eggshell fragments, and it was ensured fragments from each site were included. As noted in the text, the goal is not to confirm that all specimens are from chickens, but rather to sample enough of them to know that chickens are present and likely prominent. This does not exclude the potential that other random examples of eggshells could be found.
Data collection	Proteins were extracted from the eggshell fragments in the palaeoproteomics laboratory of the Max Planck Institute of Geoanthropology (Jena, Germany) by Carli Peters and Kristine Korzow Richter. The extracted peptide fragments were analyzed with MALDI-ToF-MS and LC-MS/MS.
Timing and spatial scale	November 2019 - September 2020 - the spacial scale covers and area of roughly 500 kilometers in diameter, as illustrated in the map in the text.
Data exclusions	No data were excluded.
Reproducibility	Samples were analyzed in triplicate and alongside a blank to make sure the data is reproducible. All attempts at reproducing the data were successful.
Randomization	Randomization was not appropriate in this study, as we simply collected all possible specimens that were permitted given the time and resources allotted.
Blinding	As we were not working with living specimens, blinding was not necessary.

Did the study involve field work? ☒ Yes ☐ No

Field work, collection and transport

Field conditions	The sample reported here have been collected over roughly 15 different field seasons, representing expeditions by many of the authors on our manuscript. The accumulation of this data has been a 7-year endeavor and it represents a wide range of difference ecological conditions from three different countries. Samples have been collected from high elevation mountains, hyper arid deserts,

	in the summer and winter, and in collaboration with different governmental organizations. In all cases, we have worked closely with representatives from each of the countries, key scholars are indicated as authors on this manuscript.
Location	Samples have been collected from 13 different archaeological sites, spanning Uzbekistan, Kyrgyzstan, and Tajikistan. The majority of the sites were in close proximity to either the modern city of Bukhara, Panjekent, or Samarkand. More specific locations are provided in the map within the text and the accompanying SI table.
Access & import/export	We have acquired all necessary import and export permits and have worked closely with local collaborators in each country. Note that these local collaborators are authors on our manuscript and they have verified that we fallowed all necessary protocols.
Disturbance	In every case that we collected samples, they were part of an ongoing archaeological excavation and the data would have been permanently lost if we had not collected it. All of this data can be considered 'rescue' archaeology. While these excavations are highly destructive to the archaeological record, the specific contribution by our study was minimal and consisted of collecting sediment samples from soil that would have otherwise been discarded of.

Reporting for specific materials, systems and methods

We require information from authors about some types of materials, experimental systems and methods used in many studies. Here, indicate whether each material, system or method listed is relevant to your study. If you are not sure if a list item applies to your research, read the appropriate section before selecting a response.

Materials & experimental systems

n/a	Involved in the study
☒	☐ Antibodies
☒	☐ Eukaryotic cell lines
☐	☒ Palaeontology and archaeology
☒	☐ Animals and other organisms
☒	☐ Clinical data
☒	☐ Dual use research of concern

Methods

n/a	Involved in the study
☒	☐ ChIP-seq
☒	☐ Flow cytometry
☒	☐ MRI-based neuroimaging

Palaeontology and Archaeology

Specimen provenance	As noted above, samples have been collected from 13 different archaeological sites, spanning Uzbekistan, Kyrgyzstan, and Tajikistan. We have worked closely with our local collaborators to obtain permission to collect the samples and in each case these collaborators worked with us to obtain export permits. Most of these key collaborators are authors on our paper, as they played important roles in ensuring that we followed all rules in the collection and exporting of the material.
Specimen deposition	The samples are currently stored at the Max Planck Institute for Geoanthropology and will remain curated with the broader project. They are accessible for future analysis.
Dating methods	Radiocarbon dates were run through Woodshole Institute. An Acid-Base-Acid (ABA) Pretreatment was used, as specified at the NOSAMS website - https://www2.whoi.edu/site/nosams/resources/methods/ All dates were calibrated using Oxcal.

☒ Tick this box to confirm that the raw and calibrated dates are available in the paper or in Supplementary Information.

Ethics oversight	We followed all ethical codes provided by the Max Planck Society and worked with local collaborators to ensure the the ethical codes of each country were followed. No independent organization reviewed the sampling protocols.

Note that full information on the approval of the study protocol must also be provided in the manuscript.
